# Assessment of burnout level among clinical dental students during the COVID-19 pandemic

**DOI:** 10.1186/s12909-023-04729-9

**Published:** 2023-10-13

**Authors:** Cumhur Korkmaz, Sibel Dikicier, Arzu Atay

**Affiliations:** Department of Prosthodontics, Hamidiye Faculty of Dentistry, University of Health Sciences, Selimiye mah. Tibbiye cad. No:38, Uskudar, İstanbul, 34668 Turkey

**Keywords:** COVID-19, Dental students, Burnout, Stress, Perceived social support

## Abstract

**Background:**

The Coronavirus Disease (COVID-19) outbreak has caused especially health workers to face mental and physical problems all over the world. The aim of this study is to evaluate burnout, stress perception and perceived social support levels of clinical (the fourth and fifth year) dental students during the COVID-19 pandemic.

**Methods:**

The Sociodemographic Information Form (SIF), Maslach Burnout Inventory (MBI), Sense of Coherence-13 (SoC-13), Perceived Stress Scale-10 (PSS-10), and Multidimensional Scale of Perceived Social Support (MSPSS) were used for data. Questionnaires were answered directly or online by the participants.

**Results:**

A total of 211 participants, 67.8% were female, 32.2% were male. Of these, 96.2% were aged 20–24 years, 84.4% were nuclear family, 11.8% were extended family, and 3.8% were blended family, and 91.5% were willingly for dentistry choice. Based on the results; the total burnout score indicated moderate burnout (41.99 ± 9.94), the SoC-13 scale indicated a strong sense of coherence (55.24 ± 7.21), the PSS-10 score (22.44 ± 3.44) indicated moderate perceived stress and the MSPSS score (65.92 ± 13.22) indicated high perceived social support. A positive correlation was found between perceived stress and burnout. Based on the personal accomplishment subscale; females, those living in extended family, and those who unwillingly choose dentistry among the clinical dental students had higher intense burnout (*p* < 0.05).

**Conclusions:**

The findings demonstrated that clinical dental students were affected by the emotional stress caused by the COVID-19 pandemic conditions. In the education of dentistry faculties, it would be appropriate to carry out comprehensive studies on adaptation to the changing living conditions with the COVID-19 pandemic and taking the necessary measures for the psychological distress caused by the difficulties experienced.

## Introduction

On December 31, 2019, a novel coronavirus causing pneumonia and quickly spread was detected in Wuhan city of China [1]. The virus was named “severe acute respiratory syndrome coronavirus-2” (SARS-CoV-2) by the World Health Organization (WHO). In March 2020, the WHO declared that COVID-19 was a global pandemic caused by this virus [2].

The first case was announced by official authorities on March 11, 2020, in Turkey [3]. With declared of the COVID-19 pandemic, many countries have implemented restrictive measures to prevent the spread of the virus, limit transmission and reduce close contact between people. Republic of Turkey Ministry of Health has also published the COVID-19 pandemic guidelines includes protection rules and treatment protocols to all healthcare workers [4]. In these guides; the importance of mask, distance and personal hygiene was emphasized, the detection of people in contact with COVID-19 disease, isolation of cases and the treatment protocol to be applied were stated [4]. The spread of the novel coronavirus through respiratory droplets and aerosols, and the contamination of blood and/or other body fluids causing cross-infection, cause those who work in the field of oral and dental healthcare to be in the high risk group [4, 5].

Evaluations such as the declaration of the pandemic and the disruption of routine life due to the measures taken, the feeling of uncertainty, the fear of being infected, and the thought of living in an unsafe area, have shown that the pandemic has both psychological effects and physiological effects [6]. Healthcare workers worldwide work under extreme stress due to excessive workload, insufficient knowledge about the mechanism of the disease, deficient personal protective equipment, fear of contracting the disease, and fear of infecting their families and relatives. All these problems negatively affect the mental health of healthcare workers [7]. In a cross-sectional study performed in China with 1257 healthcare workers, symptoms of distress were reported in 71.5%, depression in 50.4%, anxiety in 44.6%, and insomnia in 34.0% of participants [8].

Aerosols are a possible route of transmission in closed environments. Routine dental procedures pose a risk of infection for dentists, dentistry students, dental laboratory technicians, oral and dental health technicians and patients, as they generate potentially hazardous aerosols [9].

Burnout is defined as a psychological syndrome that develops against negative conditions in the workplace, and progresses with emotional exhaustion, a sense of helplessness, depersonalization, negativity towards work and life, and a decrease in personal success [10]. The burnout syndrome includes three subgroups as emotional exhaustion (EE), depersonalization (DP), and personal accomplishment (PA). Emotional exhaustion refers to lack of excitement, the feeling of exhaustion and increased stress. Depersonalization refers to emotional insensitivity to co-workers and patients. Decreased personal accomplishment associate with low self-esteem and the tendency to self-deprecation at job [10].

Healthcare workers, especially dentists, constitute a high risk group in terms of burnout syndrome due to their job responsibilities and working conditions [11]. Occupational risks such as contact with infectious agents, aspiration of droplets and aerosols, working with heavy metals, presence of noise, exposure to radiation cause an increase in the tension of dentists [11]. Additionally, their inexperience, lack of knowledge, being in the high risk group for COVID-19 infection, fear of getting sick and transmitting the disease to their relatives can cause high levels of anxiety in dentistry students [12].

In a study evaluating front-line healthcare workers during the SARS (severe acute respiratory syndrome) epidemic in Hong Kong in 2004, levels of anxiety and burnout of front-line health care workers group was found higher from control group (administrative staff), and anxiety scores and levels of burnout were found associated [13]. In study involving 2,707 healthcare workers from 60 different countries, Morgantini et al. stated that 51% reported burnout [14].

Although many studies on the effects of the COVID-19 pandemic on the knowledge, perceived stress and burnout levels of healthcare workers have been reported, there are few studies about the burnout and stress conditions of dentistry students [15, 16].

The aim of this study was to investigate the health risk concerns, burnout, stress perception and perceived social support levels of clinical (the fourth and fifth year) dental students during the COVID-19 pandemic using the questionnaire method. The null hypotheses examined in this study indicate that the COVID-19 pandemic has created a high level of stress and that there is a relationship between burnout, SoC, perceived stress and perceived social support.

## Methods

### Study design

The descriptive cross-sectional study population consisted of fourth and fifth year dental students faculties of dentistry on the Asian side of Istanbul, Turkey. The total population size was considered at a 95% confidence interval with a 0.05 margin of error in the calculated sample size using a simple random sampling method. The study protocol was approved by Institutional Ethical Board of University of Health Sciences, Istanbul, Turkey (approval number 27/16/11.12.2020).

Dentistry education in Turkey includes pre-clinic training in the first 3 years, and clinical training in which they make face-to-face applications to patients in the fourth and fifth years. Fourth and fifth year dentistry students were chosen as participants due to their exposure to both physical and psychological risks.

The inclusion criteria were: (a) being in fourth and fifth grade clinical student (b) being informed by documents and presentations about COVID-19 published by authorized organizations during the outbreak, (c) not having a history of mental illness or psychological treatment, and (d) volunteering to participate in the study. The exclusion criteria were: (a) not being informed by the documents and presentations mentioned above, (b) having a history of mental illness and psychological treatment, and (c) not volunteering to participate in the study. This descriptive cross-sectional study was conducted by paper-formed questionnaires and online with “google form” between February and May 2021.

A sociodemographic questionnaire was prepared, which included the participants’ information such as age, gender, family type and willingness to choose dentistry. Participants were informed about the purpose and benefits of the study, and a consent form was signed. Before filling out the questionnaires from the students who participated online (Google Forms), a consent form was obtained by answering the yes/no question regarding their willingness to participate in the research. Participants with incomplete or missing information and those who did not provide their consent form were not included.

### Data collection

The data of the study were collected with a 20-question sociodemographic scale, the Turkish version of the Maslach Burnout Inventory (MBI), consisting of 22 questions developed by Maslach & Jackson [10], Antonovsky’s Sense of Coherence-13 (SoC-13) [17], Perceived Stress Scale-10 (PSS-10) [18], and Multidimensional Scale of Perceived Social Support (MSPSS) [19]. The analysis of Turkish reliability and validity of the questionnaires listed below has been reported in many previous studies [17, 20–23].

### Maslach Burnout Inventory-Human Services Survey (MBI-HSS)

The survey contained 22 items with three dimensions: emotional exhaustion (EE, 9 items), depersonalization (DP, 5 items), and personal accomplishment (PA, 8 items). Each item is measured on a five-point Likert scale. The measurement of the survey is made as “Never = 0” and “Always = 4.” Scores from 0 to 32 can be obtained on the scale for the PA subscale, 0−20 for the DP and 0−36 for the EE. The burnout level of clinical dental students was used to analyze the prevalence of the following subdimensions in this study: EE (low < 18, moderate 18−19, high > 22), depersonalization (low < 10, moderate 10−11, high > 13), and PA (low < 16, moderate 16−17, high > 20). For the EE and DP subscales, higher scores signaled intense burnout, while for the PA subscale, lower scores indicated intense burnout [10]. Cronbach’s alpha was 0.87 in the current study.

### Sense of Coherence-13 (SoC-13)

According to Antonovsky [17], SoC defines how individuals can manage the diversity of stress factors in daily life. This view focuses on factors that encourage and protect well-being and resources such as capabilities and efficiencies [24]. The version used here was the SoC-13. Each item is scored on a scale of 1−7, with a total range of 13 to 91 points. A higher score indicates a powerful SoC, but no reference score has been reported in the literature. Cronbach’s alpha was 0.86 in the current study.

### Perceived stress Scale-10 (PSS-10)

The PSS-10 is a 10-item scale used to assess self-perceived stress. Each item was scored as 0, never; 1, almost ever; 2, sometimes; 3, fairly often; and 4, very often on a 5-point Likert scale. The scale forms two domains: six positively (items 1, 2, 3, 6, 9, and 10) and four negatively (items 4, 5, 7, and 8) that require reversion-worded items. Total scores range from 0 to 40, with higher scores indicating higher levels of perceived stress [18]. Cronbach’s alpha was α = 0.86 in the current study.

### Multidimensional scale of Perceived Social Support (MSPSS)

The MSPSS is a scale that consists of 12 items and rated on a 7-point Likert scale, ranging from (1) very strongly disagree to (7) very strongly agree. These items are grouped into three factors as Family (FA), Friends (FR) and Significant Others (SO). The higher the total score of the 12 items, the higher the level of perceived social support [19].

### Statistical methods

Statistical analyses of the data were conducted using IBM SPSS 20.0 for Windows (NY, USA). The total scores of MBI, SoC-13, PSS-10, and MSPSS were analyzed, and their relevant descriptive measures (mean and standard deviation [SD]) were calculated. The compatibility of the data with the normal distribution was checked with the Kolmogorov Smirnov test, and the homogeneity of the variances was checked with the Levene test. In comparison of the means between groups, t-test-ANOVA was used for normally distributed data, Mann Whitney U Test-Kruskal Wallis H test was used for non-parametric data. Pearson correlation coefficient and regression analysis were applied for the relationship between two continuous variables. Statistical significance was set at *p* < 0.05. A two-way ANOVA test was applied.

## Results

A total of 211 (98.6%) participating clinical dental students were included in the cross-sectional analysis, with consideration of completed surveys (Table 1). Of the participants, 67.8% were female, 32.2% were male. Of these, 96.2% were aged 20–24 years and 3.8% were 25–34 years. According to family type, 84.4% were nuclear family, 11.8% were extended family, and 3.8% were blended family. Most participants (91.5%) were willingly for dentistry choice.

**Table 1:** Sociodemographic characteristics of study participants (n = 211)

Characteristics	Participants (n)	Percentage (%)
**Age (year)**		
20–24	203	96.2
25–34	8	3.8
**Gender**		
Male	68	32.2
Female	143	67.8
**Family type**		
Nuclear family	178	84.4
Extended family	25	11.8
Blended family	8	3.8
**Willingness to choose dentistry**		
Willingly	193	91.5
Unwillingly	18	8.5

Regardless of sociodemographic characteristics, the total mean scores of the Turkish versions of MBI, SoC-13, PSS-10, and MSPSS were analyzed. Descriptive statistics scores along with the subgroups of the scales are given in the 95% confidence interval. According to the total burnout and subdimension scores, the total burnout score indicates moderate burnout (41.99 ± 9.94), mean EE (16.75 ± 7.47) and DP (5.17 ± 3.31) indicate low-level burnout, and mean PA sub-score (20.06 ± 4.98) indicates moderate burnout. SoC-13 scale indicates a strong sense of coherence (55.24 ± 7.21). PSS-10 score (22.44 ± 3.44) indicates moderate perceived stress. According to the MSPSS score (65.92 ± 13.22), it was defined that the level of perceived social support is high.

The relations between MBI-total, MBI subscales, SoC-13, PSS-10, MSPSS scores and gender are shown in Table 2. The differences between EE, PA and PSS-10 scores and gender were statistically significant (*p* < 0.05). No significant difference was found between males and females in the other groups.

**Table 2:** The relation between MBI-total, MBI subscales, SOC-13, PSS-10, MSPSS scores and gender

	Gender	Mean ± SD	Median (IQR)	Min-Max	%95 CI Lower-Upper	*p*
Emotional exhaustion	Male	14.87 ± 6.07	14.5 (7)	4–32	13.4-16.34	0.031*^#^
	Female	17.68 ± 7.94	17 (12)	4–36	16.36–18.99	
Depersonalization	Male	5.54 ± 3.68	5 (5)	0–15	4.65–6.44	0.42*
	Female	5.01 ± 3.11	5 (5)	0–13	4.5–5.53	
Personal accomplishment	Male	21.12 ± 5.21	21 (7)	4–34	19.86–22.38	0.03*^#^
	Female	19.51 ± 4.8	20 (6.25)	5–30	18.72–20.31	
MBI-Total	Male	41.38 ± 9.35	40 (10.75)	20–66	39.12–43.65	0.541**
	Female	42.3 ± 10.26	43 (12.25)	14–63	40.6-44.01	
SoC-13	Male	55.53 ± 7.92	55 (11)	30–72	53.61–57.45	0.686**
	Female	55.06 ± 6.88	55 (10)	39–73	53.92–56.21	
PSS-10	Male	21.66 ± 3.38	21 (3)	15–33	20.84–22.48	0.007^#^
	Female	22.8 ± 3.43	23 (4)	13–32	22.23–23.37	
MSPSS	Male	66.26 ± 11.24	66.5 (19)	40–84	63.55–68.98	0.909
	Female	65.75 ± 14.15	68 (22.25)	27–84	63.41–68.1	

*Mann Whitney U test **t test # Statistically significant (*p* < 0.05) SD-Standard Deviation, Me-Median, IQR-Interquartile range, Min-Minimum, Max-Maximum, CI-Confidence Interval of the Difference, MBI-Maslach Burnout Inventory, SoC-13-Sense of Coherence-13, PSS-10-Perceived Stress Scale-10, MSPSS- Multidimensional Scale of Perceived Social Support

Statistically significant differences were not found between MBI-total, MBI subscales, SOC-13, PSS-10, MSPSS scores and age (*p* > 0.05).

In the statistical evaluation made between MBI-total, MBI subscales, SOC-13, PSS-10, MSPSS scores and willingness to choose dentistry, it was observed that willingness to choose dentistry affects personal accomplishment (*p* < 0.05). Those who willingly chose dentistry (20.28 ± 4.91) showed lower burnout in the personal accomplishment parameter than those who unwillingly chose dentistry (17.24 ± 5.09). (Table 3).

**Table 3:** The relation between MBI total, MBI subscales, SoC-13, PSS-10, MSPSS scores and willingness to choose dentistry

	Willingness to choose dentistry	Mean ± SD	Medyan (IQR)	Min-Max	%95 CI Lower-Upper	*p*
Emotional exhaustion	Unwillingly	19.41 ± 7.75	19 (13)	9–36	15.42–23.4	0.138*
	Willingly	16.53 ± 7.44	16 (10)	4–35	15.48–17.59	
Depersonalization	Unwillingly	6.47 ± 3.34	7 (6)	1–13	4.75–8.19	0.103*
	Unwillingly	5.07 ± 3.29	5 (4)	0–15	4.61–5.54	
Personal accomplishment	Unwillingly	17.24 ± 5.09	17 (5)	7–27	14.62–19.85	0.016^#^*
	Willingly	20.28 ± 4.91	21 (7)	4–34	19.58–20.98	
MBI-Total	Unwillingly	43.12 ± 11.49	45 (15.5)	20–58	37.21–49.02	0.632**
	Willingly	41.91 ± 9.85	41 (12)	14–66	40.51–43.3	
SoC-13	Unwillingly	53.59 ± 7.68	53 (7.5)	30–65	49.64–57.54	0.334**
	Willingly	55.36 ± 7.18	55 (10.5)	39–73	54.34–56.38	
PSS-10	Unwillingly	21.82 ± 3.34	22 (4)	15–30	20.11–23.54	0.0507*
	Willingly	22.49 ± 3.46	22 (4)	13–33	22-22.98	
MSPSS	Unwillingly	61 ± 16.9	61 (30)	27–84	52.31–69.69	0.225*
	Willingly	66.35 ± 12.85	67 (21)	29–84	64.53–68.18	

* Mann Whitney U test ** t test # Statistically significant (*p* < 0.05) SD-Standard Deviation, Me-Median, IQR-Interquartile range, Min-Minimum, Max-Maximum, CI-Confidence Interval of the Difference, MBI-Maslach Burnout Inventory, SoC-13-Sense of Coherence-13, PSS-10-Perceived Stress Scale-10, MSPSS- Multidimensional Scale of Perceived Social Support

The relations between MBI-total, MBI subscales, SOC-13, PSS-10, MSPSS scores and family types are shown in Table 4. In the personal accomplishment parameter, the differences between nuclear family vs. blended family and extended family vs. blended family were statistically significant (*p* < 0.05). Additionally, the difference between nuclear family vs. extended family and extended family vs. blended family were statistically significant in terms of MBI-total score (*p* < 0.05).

**Table 4:** The relation between the MBI subscales, MBI total, SOC-13, PSS-10, MSPSS and family types

	Family type	Mean ± SD	Median (IQR)	Min-Max	%95 CI Lower-Upper	*p*
Emotional exhaustion	Nuclear family	17.16 ± 7.45	16 (11)	4–36	16.06–18.26	0.101*
	Extended family	14.08 ± 7.45	13 (7.5)	4–32	11.01–17.15	
	Blended family	16.29 ± 7.78	16 (9)	7–31	9.09–23.48	
Depersonalization	Nuclear family	5.17 ± 3.24	5 (5)	0–14	4.7–5.65	0.392*
	Extended family	4.76 ± 3.83	3 (5)	0–15	3.18–6.34	
	Blended family	7 ± 3.06	8 (6)	3–11	4.17–9.83	
Personal accomplishment	Nuclear family	20.03 ± 4.73	20 (6)	5–34	19.33–20.73	0.037^#^*^2–3^
	Extended family	19 ± 6.38	20 (8.5)	4–30	16.36–21.64	
	Blended family	23.86 ± 4.49	25 (6)	15–28	19.71–28.01	
MBI-total	Nuclear family	42.39 ± 9.6	42 (12)	14–63	40.97–43.81	0.046^#^**^1–3^
	Extended family	37.8 ± 11.99	36 (15)	18–66	32.85–42.75	
	Blended family	47.14 ± 7.45	47 (16)	37–57	40.25–54.03	
SoC-13	Nuclear family	55.25 ± 7.14	55 (10)	30–73	54.2-56.31	0.928*
	Extended family	54.88 ± 8.21	54 (14)	44–71	51.49–58.27	
	Blended family	55.43 ± 6.37	53 (12)	49–66	49.53–61.32	
PSS-10	Nuclear family	22.54 ± 3.35	22 (4)	13–32	22.04–23.03	0.215*
	Extended family	21.52 ± 3.81	21 (5.5)	16–33	19.95–23.09	
	Blended family	23 ± 4.55	23 (4)	16–31	18.8–27.2	
MSPSS	Nuclear family	66.56 ± 12.76	68 (20)	27–84	64.67–68.44	0.239*
	Extended family	64 ± 14.56	64 (28)	39–84	57.99–70.01	
	Blended family	56.57 ± 18.39	59 (29)	29–82	39.56–73.58	

* Kruskal Wallis H ** ANOVA # Statistically significant (*p* < 0.05) 1 Nuclear family vs. Extended family, 2 Nuclear family vs. Blended family, 3 Extended family vs. Blended family SD-Standard deviation, Me-Median, IQR-Interquartile range, Min-Minimum, Max-Maximum, CI-Confidence Interval of the Difference, MBI-Maslach Burnout Inventory, SoC-13-Sense of Coherence-13, PSS-10-Perceived Stress Scale-10, MSPSS- Multidimensional Scale of Perceived Social Support

According to Pearson’s correlation, a statistically significant negative correlation was found between the EE and SOC-13 and MSPSS scores, and a positive correlation was found between the PSS-10 and MBI-total scores (*p* < 0.05, r = − 0.243, *p* < 0.05, r = − 0.195, *p* < 0.05, r = 0.418, *p* < 0.05, r = 0.811, respectively). As the SOC-13 and MSPSS scores increase and, the PSS-10 and MBI-total scores decrease, the EE score decreases. A statistically significant negative correlation was found between the D and MSPSS scores, and a positive correlation was found between the PSS-10 and MBI-total scores (*p* < 0.05, r = − 0.139, *p* < 0.05, r = − 0.230, *p* < 0.05, r = 0.646, respectively). As the MSPSS score increases and, the PSS-10 and MBI-total scores decrease, the D score decreases. A statistically significant positive correlation was found between the PA and MBI-total, SoC-13 and MSPSS scores. (*p* < 0.05, r = 0.303, *p* < 0.05, r = 0.233, *p* < 0.05, r = 0.339, respectively). As the MBI-total scores decreased and, the SoC-13 and MSPSS scores increased, the PA score increased (Table 5).

**Table 5:** Correlation analysis among MBI-total, MBI subscale, SOC-13, PSS-10 and MSPSS scores

			Emotional exhaustion	Depersonalization	Personal accomplishment	MBI-Total	SoC-13	PSS-10	MSPSS
Emotional exhaustion	r	1		0.480^*^	− 0.219^*^	0.811^*^	− 0.243^*^	0.418^*^	− 0.195^*^
	*p*			0.000	0.001	0.000	0.000	0.000	0.004
Depersonalization	r	0.480^*^		1	− 0.070	0.646^*^	− 0.056	0.230^*^	− 0.139^*^
	*p*	0.000			0.311	0.000	0.418	0.001	0.044
Personal accomplishment	r	− 0.219^*^		− 0.070	1	0.303^*^	0.233^*^	− 0.085	0.339^*^
	*p*	0.001		0.311		0.000	0.001	0.221	0.000
MBI-total	r	0.811^*^		0.646^*^	0.303^*^	1	− 0.079	0.336^*^	− 0.030
	*p*	0.000		0.000	0.000		0.253	0.000	0.667
SoC-13	r	− 0.243^*^		− 0.056	0.233^*^	− 0.079	1	− 0.116	0.074
	*p*	0.000		0.418	0.001	0.253		0.092	0.287
PSS-10	r	0.418^*^		0.230^*^	− 0.085	0.336^*^	− 0.116	1	− 0.018
	*p*	0.000		0.001	0.221	0.000	0.092		0.800
MSPSS	r	− 0.195^*^		− 0.139^*^	0.339^*^	− 0.030	0.074	− 0.018	1
	*p*	0.004		0.044	0.000	0.667	0.287	0.800	

R Pearson correlation * Statistically significant MBI-Maslach Burnout Inventory, SoC-13-Sense of Coherence-13, PSS-10-Perceived Stress Scale-10, MSPSS- Multidimensional Scale of Perceived Social Support

As a result of the regression analysis, the MBI-total score (total burnout) was significantly affected by the PSS-10 score (*p* < 0.0001). The MBI-Total (total burnout) scores in females, those living in a nuclear family and those who willingly choose dentistry were significantly affected by the PSS-10 score (*p* < 0.0001) (Table 6).

**Table 6:** Regression analysis between sociodemographic characteristics and scales used

MBI-Total		R	R^2^	ANOVA	Linear Regression Equation			
					Constant	SoC-13	PSS-10	MSPSS
				p (F)	B (*p*)	B (*p*)	B (*p*)	B (*p*)
Overall		0.339	0.115	0.0001* (8.94)	24.55 (0.001)	-0.054 (0.555)	0.956 (< 0.001*)	-0.016 (0.748)
Gender	Male	0.210	0.044	0.406 (0.98)	45.887 (0.001)	-0.186 (0.209)	0.313 (0.366)	-0.015 (0.886)
	Female	0.417	0.17	0.0001* (9.74)	14.544 (0.114)	0.011 (0.927)	1.246 (< 0.001*)	-0.019 (0.731)
Age	20–24	0.332	0.11	0.000 (8.24)	23.093 (0.006)	-0.032 (0.744)	0.981 (< 0.001*)	-0.02 (0.688)
	25–34	0.875	0.76	0.095 (4.35)	21.357 (0.182)	-0.449 (0.081)	1.331 (0.023*)	0.199 (0.257)
Family type	Nuclear family	0.392	0.15	0.0001* (10.54)	25.626 (0.002)	-0.086 (0.365)	1.066 (< 0.001*)	-0.038 (0.477)
	Extended family	0.340	0.11	0.451 (0.91)	20.468 (0.377)	0.056 (0.854)	-0.17 (0.80)	0.28 (0.124)
	Blended family	0.789	0.62	0.230 (2.21)	101.69 (0.083)	-0.717 (0.237)	0.284 (0.667)	-0.379 (0.091)
Willingness to choose dentistry	Unwillingly	0.588	0.35	0.126 (2.29)	46.865 (0.083)	-0.859 (0.041*)	1.894 (0.06)	0.016 (0.925)
	Willingly	0.344	0.12	0.0001* (8.45)	19.78 (0.015)	0.014 (0.887)	0.983 (< 0.001*)	-0.011 (0.834)

* Statistically significant (*p* < 0.05)

## Discussion

In this study, it was investigated whether COVID-19 pandemic conditions can affect the burnout, stress perception and perceived social support levels of clinical dental students who work in direct contact with patients, analyzing sociodemographic variables such as age, gender, family type and willingness to choose dentistry. In our study, it was concluded that the burnout levels of clinical dental students were moderate, they managed stress well, they perceived stress at a moderate level, and their perception of social support was high due to the COVID-19 pandemic. Therefore, the null hypotheses were partly rejected.

Dentistry includes a psychologically and physically challenging education for students. Therefore, the need to have biomedical knowledge and to learn the intricacies of patient diagnosis and treatment is stressful even for motivated students planning a post-graduation career [25]. In the literature, there are many studies on the mental health of physicians and nurses during the COVID-19 pandemic [26–29]. However, there are few studies about burnout and stress perception levels of dental students during the COVID-19 outbreak [12, 16, 30]. In our study, we investigated whether the COVID-19 pandemic, which is effective all over the world, causes burnout and stress in 4th and 5th year dentistry students.

The fact that the primary symptoms of COVID-19-related outcomes (fear of contamination and stress at work) affect burnout and stress levels, suggests that the COVID-19 outbreak is an emotionally and physically stressful period. In the literature, there are different studies evaluating the stress and burnout levels between the genders. Jeong et al. [31] stated that depression and perceived stress levels were higher in men than in women, the prevalence of depression increased by 40%, and perceived severe stress increased by 30% from before the outbreak to the first year of the outbreak. In a study managed at a medicine school in China, it was demonstrated that the psychological status of female and male students was similarly affected by the COVID-19 pandemic [32]. In the study of Hakami et al. [30] with 697 dentistry students; they stated that females, students who lived alone, and junior students were more likely to experience psychological problems during the COVID-19 outbreak. Yildirim et al. [12] demonstrated that the level of anxiety was higher in females, clinical students and students who lived with an extended family during the COVID-19 pandemic. According to the outcomes of our study, the burnout and perceived stress levels in female participants were higher than those in male participants.

When the literature is examined, it has been stated that age is one of the demographic characteristics related to burnout [33]. Shrestha et al. [34] reported that there was no significant association of age with burnout among medical students in their study. Dınıbutun [35] stated that there was no significant difference between physicians in terms of total burnout levels. Additionally, Al-Rawi et al. [36] revealed that there was no significant association between burnout level and age of participants due to the COVID-19 pandemic. In another study, it was reported that the burnout level of younger healthcare workers was higher during the COVID-19 pandemic [37]. Based on the results of this study as well, no significant difference was found between the age groups evaluated in the study and the levels of burnout and stress due to the COVID-19 pandemic. It was evaluated that this result was due to the fact that most of the participants in the sample group were in the same age group.

Dentistry education encompasses a challenging educational process. The decision to choose the profession of dentistry should be made before university preferences. For this reason, we included the parameter of being willing or unwilling to choose the profession in our study.

In the studies investigating the levels of burnout due to COVID 19, no study investigating the willingness parameter in choosing a profession has been found. In our study, a significant difference was found between those who willingly chose dentistry and those who unwillingly chose dentistry, according to the personal achievement subscale. It was stated that the burnout level was lower in those who willingly choose dentistry than those who unwillingly choose dentistry. This result suggests that those who willingly choose dentistry have higher motivation for the profession and can better control the perception of burnout.

In our study, burnout, perceived stress, sense of coherence and perceived social support levels among different family types due to the COVID-19 pandemic were investigated. Based on the current findings, participants who living blended family had higher intense burnout level than participants who living nuclear family and extended family in terms of MBI-total scores. Also, participants who living extended family had higher intense burnout level than participants who living nuclear family and blended family in terms of PA scores. According to these results; It can be interpreted that the increase in burnout levels of students living in the blended family and extended family, during the COVID-19 pandemic, triggered a lack of self-confidence and increased the fear of infecting other individuals living at home.

SoC is considered an important guide to psychological well-being among individuals, indicating a source of self-protection when faced with stressful conditions [38]. Regardless of sociodemographic characteristics, it was determined that there was a strong sense of coherence between the participants. According to SoC-13 scores, all available independent variables were determined not to be significantly different from each other. According to Pearson’s correlation analysis; there was a statistically significant negative correlation between EE and SOC-13 scores (*p* < 0.05, r = − 0.243), and a positive correlation with personal accomplishment (*p* < 0.05, r = 0.233). These results show that the increase in soc levels among individuals causes a decrease in emotional exhaustion and an increase in personal achievement. Similar to our study, a previous study has shown that healthcare workers with low SoC know the effects of changing lifestyles compared with healthcare workers with moderate or potent SoC, as those with low SoC are at higher risk of poor mental health [39].

The PSS-10 is a 10-item instrument used to evaluate perceived stress level. This scale provides an understanding of how different situations affect feelings and perceived stress [18]. The current findings present moderate perceived stress levels in fourth- and fifth-year dental students, with a mean PSS-10 score of 22. In this study, the prevalence of moderate perceived stress was relatively higher than that in a previous study on Chinese healthcare workers [40]. On the other hand, the results were similar to the findings of a study in the United Kingdom [41]. However, these studies were carried out in general healthcare positions, including physicians, nurses, auxiliary health staff, and employees in management. The lack of professional experience and the younger ages of the fourth and fifth grade clinical student we investigated in our study suggest that the perceived stress level may be different.

The MSPSS is an instrument that evaluates an individual’s perception of the social support he/she receives from family, friends and significant others [19]. A high MSPSS score indicates high perceived social support. Liu et al. [42] stated that high perceived social support from family was associated with lower levels of depression and post-traumatic stress disorder. The findings of our study showed that the level of perceived social support of participants was high, and the total burnout score indicated moderate burnout. Therefore, knowing the role of high perceived social support against burnout and psychological disorders becomes even more important in epidemics such as COVID-19.

### Limitations

There were several limitations in this descriptive cross-sectional study. First, our study population consisted of fourth and fifth grade dentistry students faculties of dentistry on the Asian side of Istanbul, Turkey. We studied a relatively small sample group. In the future, researchers may study larger sample sizes. Second, the data of this study were collected through clinical student statements, so there was a remarkable risk of response bias. Moreover, as the study was not performed prospectively, the burnout and perceived stress levels of participants may have become more intense during increasingly difficult pandemic situations. Therefore, it will be important to further research the long-term effects of the COVID‐19 pandemic on the mental and physical health of front line healthcare workers.

## Conclusions

The findings showed that burnout level, SoC, perceived stress and perceived social support were important factors associated with psychological health among clinical dental students during the COVID-19 pandemic. Based on the current findings, most of the participants reported moderate burnout, SoC, perceived stress levels, and high levels of perceived social support. Especially in dentistry education, the importance of precautions to be taken against the risk of cross-infection and prevention of infectious diseases should be emphasized. The findings demonstrated that clinical dental students were affected by the emotional stress caused by the COVID-19 pandemic conditions. The reason for this condition may be due to the fear of being infected and transmitting the virus to their family members. In the education of dentistry faculties, it would be appropriate to carry out comprehensive studies on adaptation to the changing living conditions with the Covid 19 pandemic and take the necessary measures for the psychological distress caused by the difficulties experienced.

## Data Availability

The datasets used and analyzed during the current study are available from the corresponding author upon reasonable request.

## Abbreviations

COVID-19: Coronavirus Disease 2019
SARS-CoV-2: Severe acute respiratory syndrome coronavirus-2
WHO: World Health Organization
EE: emotional exhaustion
DP: Depersonalization
PA: Personal accomplishment
MBI: Maslach Burnout Inventory
SoC-13: Sense of Coherence-13
PSS-10: Perceived Stress Scale-10
MSPSS: Multidimensional Scale of Perceived Social Support

## References

[1] Chan JF, Yuan S, Kok KH (2020). A familial cluster of pneumonia associated with the 2019 novel coronavirus indicating person-to-person transmission: a study of a family cluster. Lancet.

[2] Mahase E (2020). China coronavirus: WHO declares international emergency as death toll exceeds 200. BMJ.

[3] News in Turkey. Coronavirus scientific committee meeting;2020. https://www.medimagazin.com.tr/guncel/genel/tr-sağlik-bakani-fahrettin-kocaenfekte-olan7428-saglikcalisanimiz-var-11-681-88528.html. (Accessed on 5 May 2020).

[4] COVID-19 Outbreak Management and Study Guide. 2020. https://hsgm.saglik.gov.tr/depo/covid19/ingilizce/salgin_yonetimi_ve_calisma_rehberi/covid19-salgin-yonetimi-ve-calisma-rehberi-eng.pdf. Accessed 1 June 2020.

[5] Holshue ML, DeBolt C, Lindquist S (2020). First Case of 2019 Novel Coronavirus in the United States. N Engl J Med.

[6] Kaya B (2020). Effects of pandemic on mental health. J Clin Psy.

[7] Chen S, Bonanno GA (2020). Psychological adjustment during the global outbreak of COVID-19: a resilience perspective. Psychol Trauma.

[8] Lai J, Ma S, Wang Y (2020). Factors Associated with Mental Health Outcomes among Health Care Workers exposed to Coronavirus Disease 2019. JAMA Netw Open.

[9] Ge ZY, Yang LM, Xia JJ, Fu XH, Zhang YZ (2020). Possible aerosol transmission of COVID-19 and special precautions in dentistry. J Zhejiang Univ Sci B.

[10] Maslach C, Jackson SE (1981). The measurement of experienced burnout. J Organ Behav.

[11] Castañeda Aguilera E, García de Alba García JE (2013). Análisis de los posibles factores de riesgos sociodemográficos y laborales y prevalencia del síndrome de agotamiento profesional (burnout) en odontólogos mexicanos [Analysis of possible sociodemographic and occupational risk factors and the prevalence of professional exhaustion syndrome (burnout) in mexican dentists]. Rev Colomb Psiquiatr.

[12] Yildirim TT, Atas O (2021). The evaluation of psychological state of dental students during the COVID-19 pandemic. Braz Oral Res.

[13] Poon E, Liu KS, Cheong DL, Lee CK, Yam LY, Tang WN (2004). Impact of severe respiratory syndrome on anxiety levels of front-line health care workers. Hong Kong Med J.

[14] Morgantini LA, Naha U, Wang H (2020). Factors contributing to healthcare professional burnout during the COVID-19 pandemic: a rapid turnaround global survey. PLoS ONE.

[15] Juliana SM, Josiane PS, Carla M, Luciana BO, Dayane MR, Mariane C, Graziela LC, Michele B (2022). Burnout syndrome among dentists: a systematıc review and meta-analysis. J Evid Based Dent Pract.

[16] Zarzecka-Francica EJ, Gala A, Gębczyński K, Pihut M, Wyszyńska-Pawelec G (2022). The influence of preventive activities on stress perception among Dentistry students in the period of the COVID-19 pandemic. Int J Environ Res Public Health.

[17] Antonovsky A (1987). Unraveling the mystery of health. How people manage stress and stay well.

[18] Cohen S, Kamarck T, Mermelstein R (1983). A global measure of perceived stress. J Health Soc Behav.

[19] Zimet GD, Dahlem NW, Zimet SG, Farley GK (1988). The multidimensional scale of perceived social support. J Pers Assess.

[20] Eskin M, Harlak H, Demirkiran F, Dereboy C (2013). The adaptation of the perceived stress scale into turkish: a reliability and validity analysis. New Symp J.

[21] Turkum AS (2002). The development of coping with stress scale: validity and reliabilty studies. Turkish Psychol Counseling Guidance J.

[22] Tumkaya S, Cam S, Cavusoglu I (2009). Turkish adaptation of the Burnout Snydrome Inventory short version. CU Social Sciences Institute J.

[23] Eker D, ve, Arkar H. (1995). Çok Boyutlu Algılanan Sosyal Destek Ölçeği’ nin faktör yapısı, geçerlik ve güvenirliği [Factorial Structure, Validity, and Reliability of the Multidimensional Scale of Perceived Social Support]. *Türk Psikoloji Dergisi,10*(34), 17–25.

[24] Kekäläinen T, Kokko K, Sipilä S, Walker S (2018). Effects of a 9-month resistance training intervention on quality of life, sense of coherence, and depressive symptoms in older adults: randomized controlled trial. Qual Life Res.

[25] Burk DT, Bender DJ (2005). Use and perceived effectiveness of student support services in a first-year dental student population. J Dent Educ.

[26] Zerbini G, Ebigbo A, Reicherts P, Kunz M, Messman H (2020). Psychosocial burden of healthcare professionals in times of COVID-19 - a survey conducted at the University Hospital Augsburg. Ger Med Sci.

[27] Hu D, Kong Y, Li W (2020). Frontline nurses’ burnout, anxiety, depression, and fear statuses and their associated factors during the COVID-19 outbreak in Wuhan, China: a large-scale cross-sectional study. EClinicalMedicine.

[28] Murat M, Köse S, Savaşer S (2021). Determination of stress, depression and burnout levels of front-line nurses during the COVID-19 pandemic. Int J Ment Health Nurs.

[29] Vizheh M, Qorbani M, Arzaghi SM, Muhidin S, Javanmard Z, Esmaeili M (2020). The mental health of healthcare workers in the COVID-19 pandemic: a systematic review. J Diabetes Metab Disord.

[30] Hakami Z, Khanagar SB, Vishwanathaiah S (2021). Psychological impact of the coronavirus disease 2019 (COVID-19) pandemic on dental students: a nationwide study. J Dent Educ.

[31] Jeong H, Park S, Kim J, Oh K, Yim HW (2022). Mental health of korean adults before and during the COVID-19 pandemic: a special report of the 2020 Korea National Health and Nutrition Examination Survey. Epidemiol Health.

[32] Cao W, Fang Z, Hou G (2020). The psychological impact of the COVID-19 epidemic on college students in China. Psychiatry Res.

[33] Maslach C, Schaufeli WB, Leiter MP (2001). Job burnout. Annu Rev Psychol.

[34] Shrestha DB, Katuwal N, Tamang A (2021). Burnout among medical students of a medical college in Kathmandu; a cross-sectional study. PLoS ONE.

[35] Dinibutun SR (2020). Factors Associated with Burnout among Physicians: an evaluation during a period of COVID-19 pandemic. J Healthc Leadersh.

[36] Al-Rawi NH, Yacoub A, Zaouali A (2021). Prevalence of Burnout among Dental students during COVID-19 lockdown in UAE. J Contemp Dent Pract.

[37] Tiete J, Guatteri M, Lachaux A, Matossian A, Hougardy J-M, Loas G (2021). Mental Health Outcomes in Healthcare Workers in COVID-19 and Non-COVID-19 care units: a CrossSectional Survey in Belgium. Front Psychol.

[38] Nilsson KW, Leppert J, Simonsson B, Starrin B (2010). Sense of coherence and psychological well-being: improvement with age. J Epidemiol Community Health.

[39] Zhan YX, Zhao SY, Yuan J (2020). Prevalence and influencing factors on fatigue of first-line Nurses combating with COVID-19 in China: a descriptive cross-sectional study. Curr Med Sci.

[40] Luo Y, Fei S, Gong B, Sun T, Meng R (2021). Understanding the Mediating role of anxiety and depression on the relationship between perceived stress and sleep quality among Health Care Workers in the COVID-19 response. Nat Sci Sleep.

[41] Debski M, Abdelaziz HK, Sanderson J (2021). Mental Health Outcomes among British Healthcare Workers-Lessons from the First Wave of the Covid-19 pandemic. J Occup Environ Med.

[42] Liu CH, Zhang E, Wong GTF, Hyun S, Hahm HC (2020). Factors associated with depression, anxiety, and PTSD symptomatology during the COVID-19 pandemic: clinical implications for U.S. young adult mental health. Psychiatry Res.

